# Long-term impact of juvenile idiopathic arthritis in the Greek adults' psychosocial life

**DOI:** 10.1186/1546-0096-12-S1-P191

**Published:** 2014-09-17

**Authors:** Despoina Dimopoulou, Maria Trachana, Polyxeni Pratsidou-Gertsi, George Garyfallos, Prodromos Sidiropoulos, Athina Theodoridou, Alexandros Garyfallos

**Affiliations:** 1 4th Dept of Internal Medicine, Ippokration Hospital, Greece; 2 1st Dept of Pediatrics, Pediatric Immunology and Rheumatology Referral Center, Greece; 3 2nd Department of Psychiatry , Aristotle University, Thessaloniki, Greece; 4 Dept of Rheumatology, University of Heraklion, Heraklion, Greece

## Introduction

Juvenile idiopathic arthritis (JIA) seems to have a negative impact on patients’ life style mostly due to the disease chronicity. No relevant data have been published for Greek young adults so far.

## Objectives

To capture the impact of disease burden in the psychosocial profile of adults with JIA, 17.2 years after disease onset.

## Methods

A total of 96 (66 females) patients were enrolled. Psychosocial distress was assessed by the Greek version of the self-completed paper-based General Health Questionnaire (GHQ-28). A second questionnaire regarding marital status, education level and employment status was completed by all patients. Disease activity status at the last follow-up visit was assessed according to the Wallace’s criteria, while the level of disease activity by the Disease Activity Score-28 (DAS-28). The patient’s assessment of global disease activity was measured on a Visual Analogue Scale (VAS) 0 to 10. Structural damage was scored by the Juvenile Arthritis Damage Index-Articular (JADI-A) and by the Total modified Sharp/van der Heijde Score (TmSvdHS). Physical ability was assessed by the Health Assessment Questionnaire-Disability Index (HAQ-DI).

## Results

The GHQ-28 case score depicted impaired psychosocial status in 18 patients (18.7%). The level of psychosocial distress was significantly correlated with DAS28 at the last follow-up visit (r=0.446, p<0.001). The presence of disease activity was correlated with higher degree of depression (p=0.032) and social dysfunction (p=0.008). Interestingly, patients without or with mild physical disability (HAQ-DI=0-0.49) differed from those with moderate-to-severe disability (HAQ-DI=0.5-3) in the fields of somatization (p=0.004) and social dysfunction (p<0.001), but not of depression. Higher degree of depression was recorded in the unemployed patients (p=0.018) and in those with mandatory education (p=0.018). In contrast, structural damage (JADI-A, TmSvdHS), marital status and current use or duration of corticosteroid treatment didn’t find to influence patients’ psychosocial profile. Global disease activity as rated by the patient, was found to be the only significant predictor of psychosocial distress in the multivariate analysis [B=0.057 95%CI (0.017, 0.097), P=0.005].

## Conclusion

Psychosocial distress is evident in a considerable proportion of the patients (~19%), indicating a constant impact of the disease on every-day life. The tight control of disease activity is therefore crucial in order to prevent symptoms of depression in these JIA adults.

## Disclosure of interest

None declared.

